# Taxogenomic assessment and genomic characterisation of *Weissella cibaria* strain 92 able to metabolise oligosaccharides derived from dietary fibres

**DOI:** 10.1038/s41598-020-62610-x

**Published:** 2020-04-03

**Authors:** Anna Månberger, Phebe Verbrugghe, Elísabet Eik Guðmundsdóttir, Sara Santesson, Anne Nilsson, Guðmundur Óli Hreggviðsson, Javier A. Linares-Pastén, Eva Nordberg Karlsson

**Affiliations:** 1 Biotechnology, Department of Chemistry, Lund University, PO Box 124, SE-221 00 Lund, Sweden; 2 Food Technology, Engineering and Nutrition, Lund University, PO Box 124, SE-221 00 Lund, Sweden; 3 Matís, Vinlandsleid 12, IS-113 Reykjavík, Iceland; 4 Faculty of Life and Environmental Sciences, University of Iceland, Askja, IS-101 Reykjavík, Iceland

**Keywords:** Classification and taxonomy, Genome informatics, Microbiology, Enzymes

## Abstract

The importance of the gut microbiota in human health has led to an increased interest to study probiotic bacteria. Fermented food is a source of already established probiotics, but it also offers an opportunity to discover new taxa. Four strains of *Weissella* sp. isolated from Indian fermented food have been genome sequenced and classified into the species *W. cibaria* based on whole-genome phylogeny. The genome of *W. cibaria* strain 92, known to utilise xylooligosaccharides and produce lactate and acetate, was analysed to identify genes for oligosaccharide utilisation. Clusters including genes involved in transportation, hydrolysis and metabolism of xylooligosaccharides, arabinooligosaccharides and β-glucosides were identified. Growth on arabinobiose and laminaribiose was detected. A 6-phospho-β-glucosidase clustered with a phosphotransferase system was found upregulated during growth on laminaribiose, indicating a mechanism for laminaribiose utilisation. The genome of *W. cibaria* strain 92 harbours genes for utilising the phosphoketolase pathway for the production of both acetate and lactate from pentose and hexose sugars but lacks two genes necessary for utilising the pentose phosphate pathway. The ability of *W. cibaria* strain 92 to utilise several types of oligosaccharides derived from dietary fibres, and produce lactate and acetate makes it interesting as a probiotic candidate for further evaluation.

## Introduction

In recent years there has been an increased interest in the human gut microbiota and how it is associated with health. The gut microbiota has been shown to be important for maintaining a healthy gut, for synthesis of vitamins, for proper immune function and for the metabolism^1^. With increasing understanding of the gut microbiota, the possibility to improve health by modifying its composition increases.

Probiotics are live microorganisms that, when administrated in adequate amounts, confer a health benefit on the host^2^. Mainly strains of *Bifidobacterium* and *Lactobacillus* are considered as probiotic and widely distributed for human administration, e.g. directly in capsules or incorporated in food items, such as yoghurt and juices. Other claimed probiotics include strains belonging to *Saccharomyces*, *Lactococcus*, *Enterococcus*, *Streptococcus*, *Pediococcus*, *Leuconostoc*, *Bacillus* and *Escherichia*^3^. A few strains of commensal gut microbial bacteria associated with improved host functions have also been considered probiotic^2^.

Fermented food is an attractive source of new bacteria with potential probiotic properties^4^. Expanding the research of probiotic bacteria into new strains, species and genera is important, both in order to disentangle the complex world of the gut microbiota and its effect on the host, but also to provide a proper cataloguing of probiotic taxa and perhaps, identify probiotic bacteria with novel beneficial properties.

Strains within the genus *Weissella* are interesting probiotic candidates. Bacteria within *Weissella* are Gram-positive, obligate heterofermentative lactic acid bacteria and have been isolated from various sources including faeces, saliva, breast milk and vagina of humans, as well as from plants and fermented food^5^. In previous work, four strains of *Weissella* (strains 85, 92, 142 and AV1) have been isolated from Indian fermented food and classified under the species pair *W. cibaria*/*W. confusa*^6,7^.

The isolated *W*. sp. strain 92 showed probiotic potential as it was able to utilise xylooligosaccharides (XOS) and produce lactate and acetate^7,8^. In addition, the strain survives at low *p*H and in the presence of bile acid^6^. XOS are prebiotic candidates^9^ which can be utilised by the probiotic *Lactobacillus brevis* strains and by many *Bifidobacterium* species^10,11^. Lactate and acetate can serve as nutrition source for other microorganisms in the colon, including propionate and butyrate producing bacteria^1^. Short-chain fatty acids (SCFAs), including acetate, propionate and butyrate, decrease the *p*H which inhibits pathogens and improves mineral absorption, stimulates epithelial cell growth and relieves constipation^12,13^. Butyrate stimulates proliferation of normal colon epithelial cells and prevents proliferation in colorectal cancer cells^14^.

In this study, we sequence and analyse the genomes of *W*. sp. strains 85, 92, 142 and AV1 in order to classify them into a species, a task that was not possible solely using 16 S rRNA. *W*. sp. strain 92 was subsequently chosen for further evaluation of its utilisation of prebiotic substrates and probiotic potential. For this purpose, the search for potentially prebiotic oligosaccharides that can be utilised as nutrition source was expanded, and the genome and expression of glycoside hydrolases was analysed, to understand the mechanism of degradation and utilisation of these oligosaccharides.

## Results and Discussion

### Genome sequencing necessary for species classification of *Weissella* sp. strains 85, 92, 142 and AV1

*Weissella* sp. strains 85, 92, 142 and AV1, isolated from Indian fermented food^6^ have previously been identified to belong to the species pair of *W. cibaria*/*W. confusa* by the 16 S rRNA gene sequencing method^7^. These two species are highly similar and cannot be distinguished by this method alone. Instead, the two species have been divided based on DNA:DNA hybridisation, whole-cell protein patterns, ribotyping patterns as well as different structure of the interpeptide bridge of peptidoglycan and utilisation of l-arabinose^15^. In this study, in order to classify the isolated strains, the genomes were sequenced and several approaches to whole-genome phylogeny were applied. In addition, a genomic insight into peptidoglycan synthesis and l-arabinose utilisation was gained.

Genomic DNA (gDNA) extracted from *W*. sp. strains 85, 92, 142 and AV1 was successfully sequenced, assembled into draft genomes and annotated (Table 1, Supplementary Table S1). The genomes spanned around 2.5–2.7 Mbp. For *W*. sp. strains 92, 142 and AV1, 50% of the genome was covered by two contigs and 75% by four, four and three contigs respectively. The lack of a mate pair library for *W*. sp. 85 resulted in shorter and a higher number of contigs compared to the other three strains, eight contigs were needed to cover 50% and 17 to cover 75% of the genome. The contigs of each strain were rearranged based on an alignment to the representative genome of *W. cibaria* (*W. cibaria* CH2). The alignment showed that the gaps in the genome are located at the same position in the genome for all of the assembled genomes (Supplementary Fig. S1). The numbers of coding sequences (CDS), around 2300, are in line with previous reports for *W. cibaria* and *W. confusa*, while the numbers of rRNA and tRNA genes, 12–14 and 83–86, are significantly higher^16^.

**Table 1:** Data from the assembly and annotation of the draft genomes of *Weissella* sp. strains.

	85	92	142	AV1
Total Mpb in genome	2.64	2.49	2.68	2.56
Contigs	465	84	446	196
Contigs ≥ 1000 bp	47	18	27	12
bp in largest contig	226 575	702 551	931 233	931 688
L50	8	2	2	2
L75	17	4	4	3
GC content (%)	45.44	44.85	45.11	44.89
Coding sequences	2309	2263	2298	2289
rRNA (5 S, 16 S, 23 S)	9, 2, 1	9, 2, 1	9, 2, 1	11, 1, 2
tRNA	86	83	86	85

L50 and L75 refers to the minimum number of contigs needed for covering 50% and 75%, respectively, of the genome size.

### Three phylogenetic analyses group *Weissella* sp. strains 85, 92, 142 and AV1 with *Weissella cibaria*

**Figure 1:**
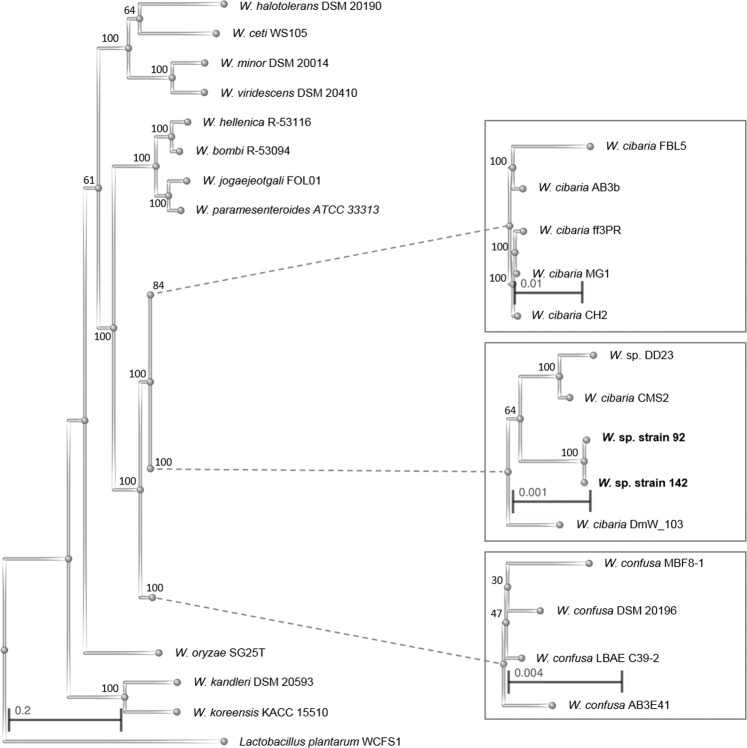
Phylogenetic tree of the genus *Weissella* based on 106 genes. The tree has a midpoint root with *Lactobacillus plantarum* WCFS1 as an outgroup. The scales represent the genetic distance as number of substitutions divided by length of the sequences. The numbers adjacent to each branch node are the bootstrap support values expressed as percentages. The amino acid sequences of the 106 analysed genes of *W. cibaria* CMS2 were identical to the corresponding genes in *W. cibaria* CMS3, *W. cibaria* CMU and *W. cibaria* KACC 11862 and the 106 analysed genes of *W*. sp. strain 142 were identical to *W*. sp. strains 85 and AV1. Thus, the latter strains were excluded from the analysis but can be expected in the same position of the tree as *W. cibaria* CMS2 and *W*. sp. strain 142, respectively.

**Figure 2:**
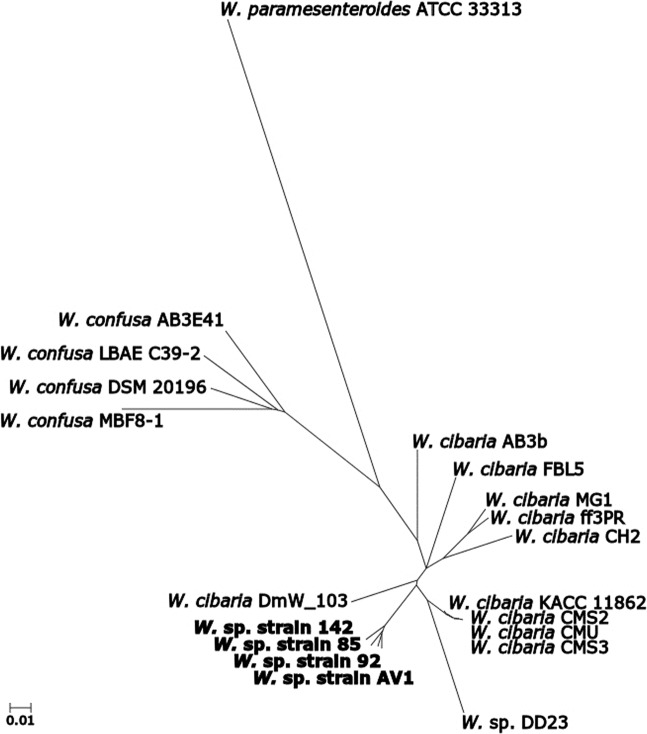
Dendrogram of strains of *Weissella cibaria* and *W. confusa, W. paramesenteroides* ATCC 33313 and *W*. sp. strains 85, 92, 142, AV1 and DD23. The dendrogram is built from a distance matrix where the distance, *d*, is measured as the part of unshared annotated genes between each pair of genomes according to Eq. (1).

Three phylogenetic analyses were done to investigate the relation of the new strains to *W. cibaria* and *W. confusa* and they all grouped *W*. sp. strains 85, 92, 142 and AV1 with *W. cibaria* (Figs. 1,2 and Table 2). Another unclassified strain, *W*. sp. DD23 was included in the analysis.

**Table 2:** Core genome of different groups within *Weissella*.

Group	Content	Core genome (%)	
		Total	Average ± SD
A	New *Weissella* sp. strains	79.1	81.8 ± 2.4
B	*W. cibaria* strains	78.8	79.3 ± 2.5
C	A + B	75.1	76.3 ± 3.5
D	*W*. sp. DD23 + B	76.4	77.3 ± 2.3
E	A + B + *W*. sp. DD23	73.2	74.7 ± 3.3
F	*W. confusa* strains	78.5	79.1 ± 1.4
G	A + F	13.8	14.2 ± 1.1
H	*W*. sp. DD23 + F	15.3	15.6 ± 0.5
I	A + B + F + *W*. sp. DD23	10.3	10.5 ± 0.6
J	H + *W. bombi* R-53094	1.7	1.8 ± 0.2
K	All *Weissella* species	< 1.0	—

Total refers to the total sequence length of the entire group and Average ± SD to average percentage of the sequences in each strain of the group ± standard deviation. Group J represent the representative genome that shared the largest core genome with the strains within group I. Group K represent Group H plus the representative genomes of all *Weissella* species deposited to NCBI.

A phylogenetic tree was constructed for all representative genomes of *Weissella*, nine and five genome sequenced strains of *W. cibaria* and *W. confusa*, respectively, and the new strains based on 106 highly conserved genes within the kingdom Bacteria (Fig. 1**)**. The new strains as well as the uncategorised *W*. sp. DD23 were grouped together with all strains of *W. cibaria*. The phylogenetic tree also shows that the new strains are more similar to each other than to the other strains of *W. cibaria*. The closest branch is the group of the *W. confusa* strains. The division of the branch between *W. cibaria* and *W. confusa* as well as the branch between the species pair *W. cibaria*/*W. confusa* and the rest of the *Weissella* species are supported 100% by 1000 bootstraps. The phylogeny (Fig. 1) groups the *Weissella* strains in four groups (1. *W. halotolerans*, *W. ceti*, *W. minor* and *W. viridescens*; 2. *W. hellenica*, *W. bombi*, *W. jogajeotgali* and *W. paramesenteroides*; 3. *W. cibaria* and *W. confusa*; and 4. *W. kandleri* and *W. koreensis*) and branches *W. oryzae* separately. The grouping is identical, with the exception of the placing of *W. oryzae*, with another phylogenetic analysis based on single-copy core orthologs of *Weissella* strains including additional *W. cibaria* and *W. confusa* strains^17^, as well as other phylogenetic analyses of the genus *Weissella* which have been based on 16 S rRNA, the *phe*S gene and DNA:DNA hybridisation^5,16^. However, the relationship between the groups differs for the different methods of analysis.

A dendrogram illustrating the part of unshared annotated genes from pairwise comparison of the genomes detecting bidirectional gene pairs covering the new strains, *W*. sp. DD23, strains of *W. cibaria* and *W. confusa*, is shown in Fig. 2. Here, the four *W. confusa* strains are clearly separated from the *W. cibaria* strains including the new strains and *W*. sp. DD23. The subdivision of the *W. cibaria* branch into two (*W. cibaria* AB3b, FBL5, MG1, ff3PR and CH2 in one and *W. cibaria* DmW_103, KACC 11862, CMS2, CMU, CMS3, *W*. sp. strains 85, 92, 142, AV1 and DD23 in the other) is also seen in the phylogenetic tree in Fig. 1. The pairwise comparison of genomes also showed that the average sequence identity of the detected bidirectional gene pairs between two species within the group of *W. cibaria* and the new strains was above 99.4% (Supplementary Fig. S2**)**, a number that shrunk below 89% for any comparison between a strain within the above-mentioned group and any *W. confusa* strain.

The size of the core genome of different groups of *Weissella* strains was calculated and presented in Table 2, and again, supports the classification of the new strains and *W*. sp. DD23 in *W. cibaria*. The core genome of the new strains was 79.1% and remained as high as 75.1% after adding the previously described *W. cibaria* strains. However, when comparing the core genome of the new strains with that of the *W. confusa* strains, the core genome decreased to only 13.8%. The corresponding numbers for *W*. sp. DD23 are 76.4 and 15.3%. A core genome phylogeny was possible by Parsnp, only for a group of strains with a core genome of above 70%. Therefore, a core genome phylogeny was constructed for the group of *W. cibaria*, the new strains and *W*. sp. DD23 and is illustrated as a cladogram in Supplementary Fig. S3. In the cladogram, *W. cibaria* AB3b, FBL5, MG1, ff3PR and CH2 are clearly grouped, as in the other phylogenetic analysis. However, as the cladogram is unrooted, the relationship within the group is more difficult to interpret. In summary, all three different phylogenetic analyses support the categorisation *W*. sp. strains 85, 92, 142, AV1 and DD23 into the species *Weissella cibaria*.

### Genes involved in biosynthesis of the interpeptide bridge of peptidoglycan reveal differences between *Weissella cibaria* and *W. confusa*

Before the advent of genome analysis, the composition of peptidoglycan was an important feature in order to classify Gram-positive bacteria^18^. The peptidoglycan structure was used to distinguish the genus *Weissella* from other genera of lactic acid bacteria as well as between species within the genus^19^. The division of *W. confusa* and *W. cibaria* is partly based on the differences in the interpeptide bridge of peptidoglycan observed by biochemical analysis where it in *W. cibaria* contains of l-Lys-l-Ala(l-Ser)-l-Ala and in *W. confusa* of l-Lys-l-Ala-l-Ala^15^. The interpeptide bridge of *W*. sp. strains 85, 92, 142 and AV1 has not been determined. However, the genomic sequences provide an opportunity to explore the interpeptide synthesis at the genomic level, comparing the new strains to *W. cibaria* and *W. confusa*.

The interpeptide bridge in peptidoglycan is catalysed by non-ribosomal peptidyl transferases that use aminoacyl-tRNA as donor substrate^20^. The genomes of *W. confusa* strains were found to contain one non-ribosomal peptidyl transferases each with unspecified activity (generic1), sharing 99% amino acid sequence identity. The strains of *W. cibaria* and *W*. sp. strains 85, 92, 142, AV1 and DD23 contain three each; one annotated as tRNA-dependent lipid II-Ala–l-alanine ligase (MurN-A), which catalyses the addition of l-alanine at the second position to l-alanine at the first position of the interpeptide bridge, and the other two with unspecific activities (generic1 and generic2). The corresponding genes in *W*. sp. strains 85, 92, 142 and AV1 were identical. In the group of *W. cibaria* and *W*. sp. strains 85, 92, 142, AV1 and DD23 the corresponding protein sequences shared 99% identity. Most similar to the generic1 protein in *W. confusa* is the generic1 protein of *W. cibaria* sharing 81% identity.

The genomic information is far from enough to explain the biochemical differences observed in the interpeptide bridge of *W. cibaria* and *W. confusa*. However, the observed difference in number of genes involved and similarity between these genes suggest a difference in the biosynthesis and is in line with the current division of the two species. Biochemical analysis is necessary to determine the interpeptide sequence of *W*. sp. strains 85, 92, 142, AV1 and DD23. However, it is evident that these strains share the set of genes involved in the biosynthesis with *W. cibaria*. Therefore, the genomic data do not reveal any reason to believe that the interpeptide sequence should be different to the one that has been observed for *W. cibaria* strains and, thus, supports the classification of *W*. sp. strains 85, 92, 142, AV1 and DD23 into the species *W. cibaria*.

### Genomic basis for arabinose utilisation found only in *Weissella cibaria* strains

**Figure 3:**
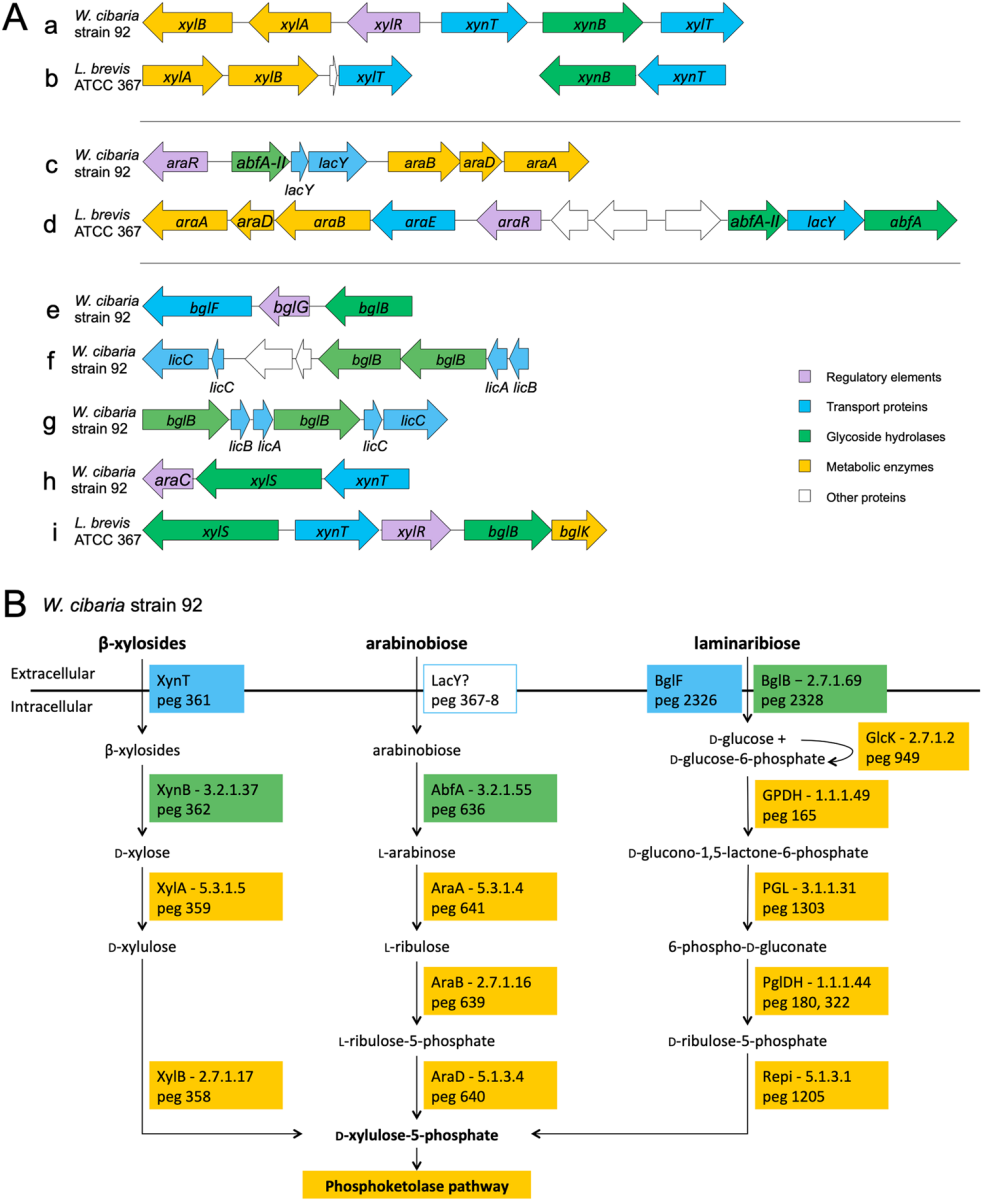
(**A**) Organisation of gene clusters in *Weissella cibaria* strain 92 and *Lactobacillus brevis* ATCC 367 involved in regulation, transport, degradation and metabolism of β-xylosides (a,b), α-l-arabinosides (c,d) and β-glucosides (e-i) including cellobiose (f-g) and α-xyloglucosides (h-i). The clusters correspond to protein encoding gene (peg) 358–363 (a), 635–641 (c), 2326–2328 (e), 298–305 (f), 1258–1263 (g) and 215–217 (h) of *W. cibaria* strain 92 annotation, and LVIS_183–186 and LVIS_2285–2286 (b), LVIS_1740–1750 (d) and LVIS_462–466 (i) of *L. brevis* ATCC 367. The genes are annotated to encode the following proteins; *xylB* – xylulose kinase, *xylA* – xylose isomerase, *xylR* – putative ROK family transcriptional regulator, *xynT* – xyloside transporter, *xynB* – β-xylosidase, *xylT* – d-xylose proton symporter, *araR* – transcriptional repressor of arabinoside utilisation operon, GntR family, *abfA-II* – α-l-arabinofuranosidase II precursor, *lacY* – lactose permease, *araB* – ribulokinase, *araD* – l-ribulose-5-phosphate-4-epimerase, *araA* – l-arabinose isomerase, *araE* – arabinose proton symporter, *abfA* – α-l-arabinofuranosidase, *bglF* – PTS, β-glucoside-specific II(**A–C**) component, *bglG* - β-glucoside bgl antiterminator bgl family, *bglB* – 6-phospho-β-glucosidase, *licA-C* – PTS cellobiose-specific IIA-C component, *araC* – transcriptor regulator, AraC family, *xylS –* α-xylosidase and *bglK* – sugar kinase (34% identity with β-glucoside kinase from *Klebsiella pneumoniae* (Uniprot Q39LQ8)). (**B**) Pathways for utilisation of β-xylosides, arabinobiose and laminaribiose based on protein activities (EC numbering) annotated within *W. cibaria* strain 92. GlcK - glucokinase, GPDH - glucose-6-phosphate 1-dehydrogenase, PGL - 6-phosphogluconolactonase, PglDH - 6-phosphogluconate dehydrogenase, decarboxylating, Repi - ribulose-phosphate 3-epimerase.

The ability to utilise l-arabinose was only found in *W. cibaria* strains and not in *W. confusa* strains when the two species were separated^15^. In the four new strains, an arabinose utilisation cluster was annotated containing transcriptional repressor of arabinoside utilisation operon, GntR family, α-l-arabinofuranosidase, lactose permease, ribulokinase, l-ribulose-5-phosphate-4-epimerase and l-arabinose isomerase, suggesting the ability to transport and metabolise l-arabinose (Fig. 3C for *W*. sp. strain 92). The same cluster was found in the genome sequenced strains of *W. cibaria* while no genes for utilisation of arabinose were found in any strain of *W. confusa*. These findings are in line with the previous observations and again support the classification of the new strains into *W. cibaria*.

*W*. sp. strains 85, 92, 142 and AV1 will from now on be referred to *W. cibaria* strains 85, 92, 142 and AV1.

### Utilisation of prebiotic oligosaccharide candidates by *Weissella cibaria* strain 92

**Figure 4:**
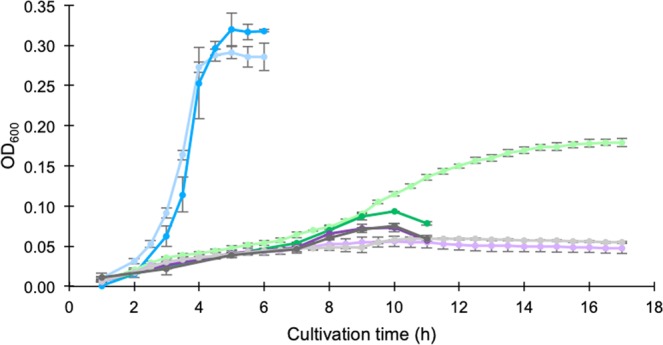
Optical density measurements at 600 nm during growth of *Weissella cibaria* strain 92 at 37 °C. Glucose (positive control, blue), cellobiose (purple) or laminaribiose (green) was used as sole carbohydrate source (2 g/L), or no carbohydrate source (negative control, grey), in microaerobic (light) or anaerobic (dark) conditions. Error bars represent standard deviation from triplicates (glucose, laminaribiose and cellobiose) or duplicates (negative control).

Utilisation of oligosaccharides derived from dietary fibre with subsequent production of short-chain fatty acids (SCFA) and lactate is a probiotic mechanism found in lactic acid bacteria. Utilisation of individual oligosaccharides varies on a strain level. *W. cibaria* strain 92 was previously found to utilise the prebiotic candidate xylooligosaccharides (XOS)^7^, a feature only observed in *Leuconostoc lactis*^21^ and a few strains of the established probiotics *Lactobacillus*^8,10,22,23^. The XOS utilisation ability of *W. cibaria* strain 92 makes it an interesting probiotic candidate. To further study oligosaccharide utilisation in the strain, screening of putative prebiotic substances (Supplementary Table S2, Fig. 4), mapping genes and pathways for oligosaccharide degradation, uptake and metabolism (Table 3, Fig. 3) and gene expression analysis of glycoside hydrolases (GHs) during growth on potential prebiotic oligosaccharides (Table 4) were performed. A genomic comparison with the probiotic *Lactobacillus brevis* ATCC 367, which, in similarity to *W. cibaria* strain 92, is known to utilise xylooligosaccharides (XOS), was made.

**Table 3:** Glycoside hydrolases annotated within the genome of *Weissella cibaria* strain 92.

Enzyme annotation	GH family	EC number	Protein encoding gene (peg) from annotation	Signal peptide	Corresponding enzyme in *L. brevis* ATCC 367, gene number – sequence identity (%)
ß-Glucosidase	GH1	3.2.1.21	197	No	—
Neopullulanase	GH13	3.2.1.135	339	No	—
Sucrose-6-phosphate hydrolase	GH32	3.2.1.26	1475	No	—
ß-Galactosidase	GH42	3.2.1.23	469	No	—
6-Phospho-β-glucosidase	GH1	3.2.1.86	302, 303, 379, 461, 905, 1258, 1261, 1679, 2328	No	465–37–60
ß-Galactosidase (small and large subunit)	GH2	3.2.1.23	1689–90	No	2258–54
Endoglucanase	GH8	—	661	Yes	1968–33
Family 13 glycoside hydrolase	GH13	—	853	No	309–40, 2180–38
α-Xylosidase	GH31	3.2.1.177	216	No	462–56
β-Xylosidase (*W*Xyn43)	GH43	3.2.1.37	362	No	2285–76, 375–32
α-l-Arabinofuranosidase (*W*Araf43)	GH43	3.2.1.55	636	No	1748–75
β-Glucuronidase	GH2	3.2.1.31	—	No	138
β-Glucosidase	GH3	3.2.1.21	—	No	1961
α-Glucosidase	GH31	3.2.1.20	—	No	137
α-Galactosidase	GH36	3.2.1.21	—	No	1758
α-N-Arabinofuranosidase	GH51	3.2.1.55	—	No	1750, 2221

**Table 4:** Relative gene expression in *Weissella cibaria* strain 92 during growth on laminaribiose and glucose determined by RT-qPCR. peg refers to protein encoding gene from annotation.

Gene name_peg	Annotation	Fold change	p-value
*bgl_197*	ß-Glucosidase	1.62	3.91·10^−3^
*bglB_302*	6-Phospho-β-glucosidase	0.316	2.68·10^−3^
*bglB_303* + *905*	6-Phospho-β-glucosidase	0.395	1.20·10^−3^
*bglB_379*	6-Phospho-β-glucosidase	0.356	2.00·10^−5^
*bglB_461*	6-Phospho-β-glucosidase	0.871	2.50·10^−1^
*bglB_1258*	6-Phospho-β-glucosidase	0.660	3.98·10^−2^
*bglB_1261*	6-Phospho-β-glucosidase	0.913	3.32·10^−1^
*bglB_1679*	6-Phospho-β-glucosidase	13.9	1.48·10^−4^
*bglB_2328*	6-Phospho-β-glucosidase	63.5	2.57·10^−5^
*eng_661*	Endoglucanase	0.715	3.34·10^−3^
*gh13_853*	Family 13 glycoside hydrolase	3.49	1.22·10^−3^
*bglB_303*	6-Phospho-β-glucosidase	0.305	4.81·10^−4^

### Xylooligosaccharides

A XOS degrading cluster was annotated in the genome of *W. cibaria* strain 92 (Fig. 3). *W. cibaria* strain 92 was previously shown to utilise xylobiose (X_2_), xylotriose (X_3_) and xylotetraose (X_4_)^7^, and a GH family 43 (GH43) β-xylosidase (*W*Xyn43) with high activity on X_2_ and X_3_ and reduced activity on X_4_, has been characterised^24^. The gene encoding *W*Xyn43, corresponding to protein encoding gene (peg) 362 was found in the genome in a cluster containing genes annotated to a xylose-responsive transcriptor regulator, a xyloside transporter and activities for further metabolism by conversion of d-xylose via d-xylulose to d-xylulose-5-phosphate (Fig. 3).

The gene organisation for XOS utilisation differs from *L. brevis* ATCC 367. *L. brevis* ATCC 367 harbours two annotated GH43 β-xylosidases, LbX with 76% identity (LVIS_2285) and another potential β-xylosidase with 32% identity (LVIS_375) to *W*Xyn43. A xyloside transporter was found downstream of LbX while the genes for metabolism are placed elsewhere in the genome (Fig. 3). *W*Xyn43 was found to hydrolyse X_3_ more effectively than LbX which has a reduced activity on X_3_ compared to X_2_^25^.

### Arabinooligosaccharides

Arabinobiose (A_2_) utilisation by *W. cibaria* strain 92 was observed during screening of prebiotic substrates (Supplementary Table S2). Arabinooligosaccharides (AOS), consisting of α-1,5 linked l-arabinofuranoside resides, are degradation products from the polysaccharide arabinan found in e.g. sugar beet, soy bean, quinoa, apples, carrot, pears and cabbage. Cultivation of *W. cibaria* strain 92 on A_2_ as sole carbohydrate source resulted in an increase in optical density (OD), decrease in *p*H and production of lactate, acetate and trace amounts of butyrate. No growth or production of lactate or SCFAs was detected for the longer AOS with a degree of polymerisation (DP) of 3–5. Utilisation of AOS was expected due to presence of the previously characterised GH43 α-l-arabinofuranosidase from *W. cibaria* strain 142 (*W*Araf43)^26^, which has an identical sequence to the corresponding protein in *W. cibaria* strain 92 (peg 636). *W*Araf43 in *W. cibaria* strain 92 was found in a cluster including genes annotated to a transcriptional repressor, a lactose permease and three genes for the conversion of l-arabinose into d-xylulose-5-phosphate, via l-ribulose and l-ribulose-5-phosphate (Fig. 3). Interestingly, the *W*Araf43 enzyme showed activity on both A_2_ and A_3_^26^ while *W. cibaria* strain 92 did not grow on A_3_ (Supplementary Table S2). This suggests that there is a transportation limitation over the cell membrane for AOS with a DP above 2 as *W*Araf43 lacks a signal peptide and is thus expected to be intracellular.

*L. brevis* ATCC harbours an annotated α-l-arabinoside utilisation locus which is larger and constructed differently compared to the corresponding cluster in *W. cibaria* strain 92, additionally including an arabinose proton symporter and one GH51 α-N-arabinofuranosidase (LVIS_1750) (Fig. 3). The GH43 α-l-arabinofuranosidase (LVIS_1748) has 75% identity to *W*Araf43. *Lb*Araf43, a homolog to LVIS_1748, from *L. brevis* DSM 1269 has been shown to have similar activity to *W*Araf43, active on AOS with a DP of 2–3 but not on α-1,3-linked l-arabinofuranosides in arabinoxylooligosaccharides (AXOS)^26^. *L. brevis* ATCC also has a third α-l-arabinofuranosidase, belonging to GH51 (Table 3). *L. brevis* ATCC 14869 has been shown to utilise linear arabinooligosaccharides with subsequent production of acetate and lactate, a feature that was not conserved within the genus of *Lactobacillus*^27^.

### β-Glucosidic oligosaccharides

*W. cibaria* strain 92 grows on laminaribiose under microaerobic conditions. During microaerobic growth on laminaribiose, the OD reached 60% of the OD obtained at growth on glucose at the same concentration (Fig. 4), while no growth was observed for longer laminarioligosaccharides (Supplementary Table S2). Only minor growth was detected on laminaribiose under anaerobic conditions (Fig. 4). The growth on laminaribiose was slow compared to growth on glucose (Fig. 4) indicating the need of induction of one or several genes for its utilisation. The expression of all GHs annotated in the genome potentially involved in degradation of β-glucosidases was analysed by real-time quantitative reverse transcription-PCR (RT-qPCR) during growth of *W. cibaria* strain 92 on laminaribiose and glucose (Table 4). Two genes annotated as 6-phospho-β-glucosidases, peg 1679 and 2328, were found to be significantly upregulated during growth on laminaribiose compared to glucose. The strongest upregulation was found for peg 2328 which is clustered with a β-glucoside-specific phosphotransferase system (PTS) and a β-glucoside antiterminator indicating the mechanism for uptake and hydrolysis of laminaribiose into d-glucose and 6-phospho-d-glucose for further metabolism in the cell (Fig. 3).

Laminarioligosaccharides originate from laminarin, a mainly β-1,3-linked polymer found in brown seaweeds^28^. Related polymers are however also produced by many bacterial species, including curdlan, an insoluble polymer produced by *Agrobacterium* sp.^29^, and β-1,3-linkage containing exopolysaccharides (EPS) produced by *Pediococcus parvulus*^30^ and *Lactobacillus brevis*^31^. The β-1,3-linked disaccharide laminaribiose, can also originate from mixed-linkage β-glucans, e.g. from cereals, such as rice and oat. *W. cibaria* strain 92 has been isolated from idli batter, an Indian fermented dish made from rice and lentils.

The prebiotic potential of laminaribiose has recently been addressed together with that of other oligosaccharides derived from curdlan^32^. They were found to fulfil the prebiotic criteria of resisting host digestion and being fermented by intestinal bacteria *in vitro*. The stimulation of *Lactobacillus*, *Bifidobacterium* and *Bacteroides* with laminaribiose increased production of acetate, propionate and lactate and decreased the *p*H. The ability to utilise laminaribiose by *Lactobacillus* was found to be strain dependent with most tested strains able to utilise it, the exception being *L. brevis* NRRL B-4527. *L. brevis* ATCC 367 possesses only one 6-phospho-β-glucosidase annotated in the genome, which is not located in proximity to a PTS. The PTS is instead included in a cluster including an α-xylosidase, a xyloside transporter and a sugar kinase (Fig. 3) indicating a putative role during utilisation of xyloglucan-oligosaccharides. However, its specificity has not been determined.

The ability of *W. cibaria* strain 92 to grow on laminaribiose might also originate from the ability to hydrolyse and metabolise self-synthesised oligo- or polysaccharides. Three putative β-1,3-glucotransferases from glycosyltransferase family 2 (GT2) lacking signal peptides were annotated in the genome. The β-1,3-glucotransferase activity implies that the bacterium can synthesise carbohydrates including this linkage. No characterised homologues were found during protein BLAST search, hence, the activity is unestablished, and the native role of 1,3-glucoside metabolism remains a hypothesis. *W. cibaria* strains 85, 92, 142 and AV1 are known to produce EPS during growth on sucrose^6^. The linkage type of the produced EPS has however not been investigated, and EPS including β-1,3-glucosidic linkage has not previously been reported for *Weissella*, while it has for other lactic acid bacteria including *L. brevis* TMW 1.2112. Interestingly, *L. brevis* TMW 1.2112 has a GT2 involved in the EPS formation as well as a homologue to the putative extracellular GH8 β-glucanase of *W. cibaria* strain 92, suggested to be involved in degradation of the EPS^31^. EPS from other strains of *W. cibaria* are reported to be dextran-like polysaccharides with varying composition between strains regarding α-1,6 and α-1,3 linkages as well as presence of other monosaccharides^33–35^. As EPS can act as a prebiotic substance in itself, the structure of the EPS produced under different conditions is of interest in a probiotic context, but out of the scope of this study.

Surprisingly, *W. cibaria* strain 92 did not grow on cellobiose, neither in microaerobic nor in anaerobic conditions (Fig. 4). Growth on cellobiose was expected due to the presence of cellobiose-specific PTS in the genome. Several PTS in proximity to one or two 6-phospho-β-glucosidases are annotated in the genome, two of which are complete with A, B and C components and categorised as cellobiose-specific (Fig. 3). This implies either non-optimal conditions used for growth on cellobiose, or an over-annotation of cellobiose-specific PTS. Besides cellobiose-specific PTS, the genome also harbours annotated genes for complete PTS specific for *N*-acetylglucosamine, sucrose, fructose, mannose and β-glucosides as well as components B and C of PTS specific for arbutin, cellobiose, and salicin, and trehalose. PTS are common in *Lactobacillus*^36^, however, the number is not that high in *L. brevis* ATCC 367, which only has one complete annotated PTS for mannose and the C component of PTS specific for galactitol and cellobiose. *L. brevis* ATCC 367 can utilise cellobiose^37^ and its genome encodes an intracellular GH3 β-glucosidase lacking a homologue in *W. cibaria* strain 92 (LVIS_1961, Table 3). *L. brevis* ATCC 367 has been shown to hydrolyse cellobiose^38^ suggesting another transportation mechanism than PTS. Clearly, annotation of PTS is weak. This could be due to a low conservation of the primary sequence and lack of experimental data^39^. Biochemical investigation of such must be applied before conclusions can be made.

In summary, like many strains of *Lactobacillus*, *W. cibaria* strain 92 utilises laminaribiose. This is however, to our knowledge, the first report of utilisation in a lactic acid bacteria that has been coupled with gene upregulation, in this case two 6-phospho-β-glucosidases, one of them in proximity to a PTS. Laminaribiose utilisation has not been observed in strains of the XOS utilising *L. brevis*. Cellobiose utilisation could not be detected in *W. cibaria* strain 92 while its utilisation in strains of *L. brevis* has been established.

### Production of lactate and SCFAs in *Weissella cibaria* strain 92

*W. cibaria* strain 92 can produce lactate, acetate, ethanol and minor amounts of butyrate from fermentation of various monosaccharides and oligosaccharides (Supplementary Table S2**)**. During screening of different substrates, lactate was detected in all samples where growth had occurred. Acetate was detected in all samples except when the bacteria had fermented glucose in microaerophilic conditions and uptake of acetate from the growth medium had occurred. Ethanol was only detected in samples with fermentation of hexoses. Butyrate was only detected in minor amounts in samples where fermentation of pentoses had occurred. Propionic acid was not detected in any sample.

*W. cibaria* strain 92 is heterofermentative and utilises the phosphoketolase pathway for metabolising carbohydrates into lactate, acetate and ethanol. All genes necessary in the phosphoketolase pathway for production of three products from the monosaccharides d-glucose, l-arabinose and d-xylose are annotated in the genome. The same set of genes were also found in *L. brevis* ATCC 367, where also transketolase (EC 2.2.1.1) and transaldolase (E.C. 2.2.1.2) were annotated, enabling the use of the pentose phosphate pathway resulting in lactose only. While grown on partly hydrolysed xylan, *W. cibaria* strain 92 produced around the same amounts of lactate and acetate while *L. brevis* DSMZ 1269 produced more lactate than acetate^8^.

Trace amounts of butyrate were detected in the media during growth of *W. cibaria* strain 92 with the pentoses arabinose and xylose under strictly anaerobic condition and with A_2_ during microaerobic growth conditions in cuvettes (Supplementary Table S2**)**. No butyrate was detected during growth on any hexoses.

The main butyrate pathways reported for butyrate producing bacteria in the colon include the conversion of acetyl-CoA to butyrate via crotonyl-CoA and butyryl-CoA^40^. Amino acids such as glutarate, lysine and 4-aminobutyrate, act as starting substrate for the synthesis, joining the acetyl-CoA pathway from crotonyl-CoA and downstream to butyrate. Necessary for these pathways is butyryl-CoA dehydrogenase (including electron transfer protein α-and β-subunits) catalysing crotonyl-CoA into butyryl-CoA (EC 1.3.8.1). No genes in the genome of *W. cibaria* strain 92 can be found encoding the mandatory butyryl-CoA dehydrogenase. Only one gene potentially involved in the butyrate synthesis pathway reported in the literature was found in the genome. The activity for catalysing the last step in the acetyl-CoA pathway, EC 2.8.3.8, was annotated for one gene, peg 543. In several butyrate producing bacteria, this gene has been observed at a different genetic location than the other genes involved in butyrate synthesis, which have been clustered together^41^.

Recently, Botta and co-workers proposed a new pathway for butyrate synthesis in *L. plantarum*, involving the fatty acid synthesis type II (FASII) pathway and a medium-chain thioesterase^42^. In this pathway, acetyl-CoA is converted to malonyl-ACP which enters the cycle of fatty acid elongation where the fatty acid chain is elongated by two carbons in each cycle. The cycle can be erupted by acetyl-ACP thioesterase (EC 3.1.2.14) which cleaves acyl-ACP and releases ACP and the fatty acid which could have a chain length of C4 (i.e. butyrate), C6, C8, C10, C12 or C14. This is a potential butyrate synthesis pathway in *W. cibaria* strain 92 as all genes involved in this pathway except one have been annotated. Acyl-carrier-protein synthase (ACPS), E.C. 2.7.8.7 has not been annotated in the genome. In both *L. brevis* ATCC 367 and *L. plantarum* WCSF1, ACPS is positioned upstream of an alanine racemate. The annotated homologous alanine racemate in *W. cibaria* strain 92 (peg 139) contains the sequence for both ACPS and the alanine racemate. The sequence for ACPS has 47 and 46% identity to the ACPS from *L. brevis* ATCC 367 and *L. plantarum* WCSF1, respectively. This possible butyrate producing pathway is worth investigating further.

Butyrate is the preferred energy source for colon epithelial cells and has shown many positive effects on its host when present in the colon, including stimulating proliferation of normal colon epithelial cells and prevention of proliferation in colorectal cancer cells, besides the positive effect described for SCFAs in general^14^. Further studies aiming at expanding the knowledge of the butyrate-producing capacity of *W. cibaria* strain 92, including the identification of substrates stimulating the production and understanding of the metabolic pathway is of high interest and important for the probiotic potential.

## Conclusions

*Weissella* sp. strains 85, 92, 142 and AV1 have been genome sequenced and classified into the species *W. cibaria* by whole-genome phylogeny. *W. cibaria* strain 92 is heterofermentative and produces lactate and acetate from the phosphoketolase pathway and can produce ethanol during growth on hexose sugars and small amounts of butyrate during growth on pentose sugars. *W. cibaria* strain 92 can grow on a variety of oligosaccharides derived from dietary fibres including xylooligosaccharides, arabinobiose and laminaribiose and the gene clusters including genes for transportation, degradation to monosaccharides and metabolism have been identified by annotation of the sequenced genome. The glycoside hydrolyses of the gene cluster responsible for degradation of xylooligosaccharides and arabinobiose have previously been characterised while upregulation of two 6-phospho-β-glucosidases during growth on laminaribiose was shown in this study. The genome of *W. cibaria* strain 92 contains a large number of phosphotransferase systems with unknown specificity and glycoside hydrolases potentially hydrolysing β-glucosides, α-xylosides, β-galactosides and α-glucosides.

## Experimental Procedures

### Preparation of DNA library

*Weissella* sp. strains 85, 92, 142 and AV1 have previously been isolated from dahi, idli batter, cucumber and fermented cabbage, respectively^6^.

*W*. sp. strains 85, 142 and AV1 were cultured in liquid MRS medium (Merck, Darmstadt, Germany) in an anaerobic environment, at 37 °C for 48 h. The liquid cultures were centrifuged at 3076 × g for 5 min and the cell pellets were used to extract genomic DNA (gDNA) using the MasterPure Gram-positive DNA Purification Kit (Epicentre-Illumina, San Diego, CA) according to the manufacturer’s instructions. DNA concentration and purity were assessed using the Qubit dsDNA BR Assay Kit (ThermoFisher Scientific, Waltham, MA) and NanoDrop. The quality of the extracted DNA was analysed on a 1.0% TAE-Agarose gel. *W*. sp. strain 92 was grown as described below. For each strain, the extracted DNA was used as starting material for the preparation of a DNA library with Illumina TruSeq indexes (Illumina, San Diego, CA). For *W*. sp. strains 85, 142 and AV1, libraries were made using the TruSeq Nano method according to the manufacturer’s guidelines, and for *W*. sp. strain 92, the library was made using the TruSeq PCR-Free method, performing fragmentation of gDNA by Fragmentase digestion (NEBNext dsDNA Fragmentase, used as recommended in the product protocol) and according to the Illumina protocol from end-repair and size-selection onwards.

### Preparation of mate pair library

*W*. sp. strains 92, 142 and AV1 were cultured in 90 mL liquid MRS growth medium (Merck, Darmstadt, Germany). The cultivation took place in an anaerobic environment, at 30 °C for 48 hours. The liquid cultures were centrifuged at 3076 × *g* for 5 min. Supernatants were discarded and the cell pellets were used as starting material for DNA-extraction with the ZR Fungal/Bacterial DNA MiniPrep kit (Zymo Research, Orange, CA) which was performed according to the supplier’s instructions with minor adaptations. The quality of the extracted DNA samples was analysed on a 0.6% agarose gel and the quantities were determined by NanoDrop and Qubit.

For each strain, the extracted DNA was used as starting material for the preparation of a mate pair library with Nextera Mate Pair Library Prep, Gel-Free method (Illumina, San Diego, CA). The procedure followed the manufacturer’s instructions with some exceptions. Purifying the tagmentation reaction (Step 1 to 8) was done by NucleoSpin gDNA clean-up kit (Macherey-Nagel, Düren, Germany) according to the accompanying protocol. Shearing of circularised DNA was done on a Covaris M220, each sample divided in three aliquots and with the following settings to match the original procedure; peak power intensity: 75 W, duty cycle/duty factor: 20%, cycle per bursts: 200, time: 50 s and temperature: 6 °C.

### Sequencing

Both the DNA and mate pair libraries were sequenced on the Illumina MiSeq system using the V3 chemistry with 2x300bp read lengths for the sequencing of the DNA libraries and the V2 chemistry with 2x250bp read lengths for the mate pair sequencing. Data on number of reads and coverage is presented in Supplementary Table S1.

### Genome assembly

Sequencing data from both the DNA and mate pair libraries were used for genome assembly. For *W*. sp. strain 85, only a DNA library was available and consequently used. Before assembly, the reads from the sequencing were trimmed and filtered based on quality by the software Trimmomatic version 0.36^43^. For DNA library reads, the following settings, in this sequence, were chosen in order to optimise recovery of reads and quality: illuminaclip:truseq. 3-pe.fa:2:30:10, leading:3, trailing:3, crop:275, headcrop:20 and avgqual:30. For the mate pair reads, the same settings were applied with the exception of crop:245. A quality control of the output reads was done by FastQC version 0.11.5^44^. All reads resulted as “entirely normal” or “slightly abnormal” for all quality parameters with the exception of “kmer content” and “per tile sequence quality” which for a minority of the reads, mainly from the DNA libraries, resulted in “very unusual”. The recovery of reads and coverage after the trimming and quality filtering are presented in Supplementary Table S1.

The trimmed and filtered reads for each strain were assembled using SPAdes version 3.7.0^45^. Both the paired reads and unpaired reads of the DNA and mate pair libraries were used as input for the genome assembly^46,47^. Quality assessments of the assembled genomes were produced by the QUAST (Quality Assessment Tools for Genome Assemblies) web interface (http://quast.bioinf.spbau.ru/)^48^. The assemblies resulted in draft genomes containing several contigs. Data from the assemblies are presented in Table 1.

The contigs of the assembled draft genomes were reordered and renumbered relative to the complete genome of *W. cibaria* CH2, downloaded from National Center for Biotechnology Information (NCBI) genome database (ftp://ftp.ncbi.nih.gov/genomes/)^49^, in Mauve version 2015–02–25^50,51^. A multiple sequence alignment of the reordered genomes was done in Mauve to visualise rearrangements between the genomes and can be seen in Supplementary Fig. S1.

### Genome annotation

The reordered assembled draft genomes were added to the RAST Server (Rapid Annotation using Subsystem Technology) (http://rast.nmpdr.org/)^52^ for annotation. Gene numbers presented in the paper are according to the numbering done by the RAST server. Number of rRNA and tRNA genes were predicted by RNAmmer 1.2 (http://www.cbs.dtu.dk/services/RNAmmer/)^53^ and tRNAscan-SE 2.0 (http://lowelab.ucsc.edu/tRNAscan-SE/)^54^, respectively, both accessed through the corresponding web interface.

### Phylogeny of *Weissella* sp. strains based on 106 highly conserved genes

A phylogenetic tree of the genus *Weissella* was constructed by the bcgTree pipeline^55^ from, within the kingdom Bacteria, 106 highly conserved genes. Proteomes from the representative genomes of all *Weissella* species and nine and five strains of *W. cibaria* and *W. confusa*, respectively, available in the NCBI genome database as well as the proteome of the representative genome of *Lactobacillus plantarum* were downloaded in the amino acid FASTA format (*.faa). Proteomes from *W. cibaria* FBL5, *W. cibaria* KACC 11862 and *W. halotolerans* DSM 20190 were not available from the NCBI Genome database. In these cases, the genomes were downloaded in the nucleotide FASTA file format (*.fna) and added to the RAST server for annotation. The *L. plantarum* and *Weissella* proteomes together with the proteomes obtained from the RAST server of *W*. sp. strains 85, 92, 142 and AV1 were used as input files for the bcgTree pipeline. The tree was calculated with 1000 bootstraps and visualised by the NCBI’s Tree Viewer application (https://www.ncbi.nlm.nih.gov/projects/treeview/) as a rectangle cladogram with a midpoint root.

### Distance based tree of gene content of *Weissella cibaria* and *Weissella confusa*

A dendrogram of *W*. sp. strains 85, 92, 142, AV1 and DD23, strains of *W. cibaria* and *W. confusa* and *W. paramesenteroides* ATCC 33313 was constructed from a distance matrix of gene content. Genomes from all available strains of *W. cibaria* and *W. confusa*, *W*. sp. DD23 as well as the representative genome of *W. paramesenteroides* were downloaded from the NCBI Genome Database and added to the RAST Server for annotation. Pairwise comparison of all proteomes detecting gene pairs by sequence-based comparison of the annotated genes was done by the SEED Viewer of the RAST server (http://rast.nmpdr.org/seedviewer.cgi)^56^. The SEED Viewer marked each gene pair as unidirectional or bidirectional and calculated the sequence identity. A distance matrix was constructed based on the bidirectional gene pairs in each pair of genomes. The distance, *d*, between the genomes *G*_*1*_ and *G*_2_ was calculated according to Eq. (1),

1 $$d({G}_{1},{G}_{2})=1-\left(\frac{|{G}_{1}{\cap }^{}{G}_{2}|}{{\rm{\min }}(|{G}_{1}|,|{G}_{2}|)}\right),$$

where *│G*_1_* ∩ G*_2_*│* is the number of bidirectional gene pairs in *G*_*1*_ and *G*_*2*_ and *│G*_*i*_*│* is the total number of annotated genes in *G*_*i*_^57,58^. The distance method neighbour-joining^59^ was applied to calculate the phylogenetic tree from the distance matrix in the T-REX (Tree and Reticulogram Reconstruction) web server^60^. The output tree was visualised by the tree viewer of T-REX web server (http://www.trex.uqam.ca/) in the radial format.

### Core genome size and phylogeny

Parsnp version 1.2^61^ was used to determine the size of the core genome for various groups of *Weissella* strains. The nucleotide fasta files (*.fna), obtained from the NCBI genome data bank or from the genome assemblies, were used as input data. The group of *W. cibaria* strains and *W*. sp. strains 85, 92, 142, AV1 and DD23 were considered to be a group of closely related strains with a core genome large enough for generation of a whole-genome phylogenetic analysis. Parsnp was used to construct a phylogenetic tree with *W*. sp. strain 92 as reference genome. The phylogenetic construction was visualised by NCBI’s tree viewer as an unrooted rectangle cladogram.

### Analysis of genes involved in formation of the interpeptide bridge of peptidoglycan

Genes involved in the formation of an interpeptide bridge in peptidoglycan were found in the RAST-server generated subsystem “tRNA-dependent amino acid transferases” through the SEED Viewer. The annotated proteins within the genome sequenced strains of *W. cibaria* and *W. confusa*, *W*. sp. DD23 as well as *W*. sp. strains 85, 92, 142, AV1 were compared by protein BLAST from NCBI’s web interface (https://blast.ncbi.nlm.nih.gov/Blast.cgi)^62^.

### Analysis of genes and pathways involved in carbohydrate utilisation in *Weissella* sp. strain 92

Genes and pathways involved in degradation, uptake and metabolism of oligosaccharides and production of lactate and SCFAs were found in the RAST-server generated subsystems and by manual search by EC numbering. Glycoside hydrolases (GHs) with unannotated activity were compared with protein data banks by protein BLAST from NCBI’s web interface^62^. Signal peptides were searched for by SignalP 4.1 (http://www.cbs.dtu.dk/services/SignalP-4.1/)^63^. Comparison with the genome of *Lactobacillus brevis* ATCC 367^64^ was done based on the publicly available RAST annotation as well as protein BLAST search in the genomes of genes only annotated in one of the two organisms.

### Screening for carbohydrate utilisation in *Weissella* sp. strain 92

*W*. sp. strain 92 was cultivated in MRS medium (10 g/L casein peptone tryptic digest, 8 g/L meat extract, 4 g/L yeast extract, 1 g/L Tween 80, 2 g/L K_2_HPO_4_, 5 g/L Na-acetate · 3H_2_O, 2 g/L (NH_4_)_3_-citrate, 0.2 g/L MgSO_4_ · 7H_2_O, 0.05 g/L MnSO_4_ · 4H_2_O and 20 g/L carbohydrate source). The monosaccharides d-glucose, l-arabinose, d-xylose and *N*-[1] acetylglucosamine, and the oligosaccharides arabinobiose (A_2_), arabinotriose (A_3_), arabinotetraose (A_4_), arabinopentaose (A_5_), laminaribiose, laminaritriose, laminaritetraose and diacetyl chitobiose (Megazyme, Wicklow, Ireland) were filter sterilised by a 0.2 µm filter, autoclaved and added to the medium as carbohydrate source.

Cultivations in cuvettes using 1 mL medium were done with glucose, arabinose, *N*-acetyl glucosamine, the arabinooligosaccharides (AOS), the laminarioligosaccharides and diacetyl chitobiose as the respective carbohydrate source. Cultivations in flasks using 75 mL medium with glucose, xylose and arabinose as the respective carbohydrate source, as well as a negative control without any added carbohydrates were incubated anaerobically. All cultivations were incubated at 37 °C for 48 h. The cell density was monitored by optical density (OD) measurement at 600 nm. The 1 mL cultures were filtrated by a 0.2 µm sterile filter. The 75 mL cultures were centrifuged at 13,000 *g* for 10 min, following separation of the supernatant. *p*H was measured on the filtrates as well as the medium before cultivation.

### Analysis of SCFAs, lactic acid and ethanol by HPLC

The content of SCFAs, lactic acid and ethanol was analysed in the filtrates and supernatants by HPLC coupled with a RI-detector (Ultimate 3000 RSLC, Thermo Fisher Scientific, Waltham, USA). 10 µl of each sample was injected and separated on an ion exchange column (Aminex HPX-87H) at 40 °C with a mobile phase of 0.5 mM H_2_SO_4_ at a flow rate of 0.5 mL min^−1^. Standards used were acetic acid, butyric acid, lactic acid, propionic acid and ethanol.

### Growth curves

Growth curve experiments for *W*. sp. strain 92 on laminaribiose, cellobiose and glucose, and without a carbohydrate source (negative control) were conducted in anaerobic and microaerobic conditions. All cultivations were performed in triplicate, except the negative controls which were done in duplicate. MRS medium was prepared as previously described except that 2 g/L of the carbohydrate sources was used. Cultivations of 20 mL were grown anaerobically at 37 °C after inoculation of colonies grown on MRS agar medium over-night. Samples of about 200 µl were taken at different time points, 150 µl was transferred to a 96-well plate and OD was measured at 600 nm in a microplate reader. Cultivations of 250 µl were grown in microaerobic conditions at 37 °C in a 96-well plate with lid on after inoculation of 2% (v/v) cells grown in MRS medium over-night. The OD was measured at 600 nm in a microplate reader at different time points. All absorbance values from the microplate reader were adjusted to correspond to a measurement length of 1 cm.

### Real-time quantitative reverse transcription-PCR

Cultivations of 5 mL of *W*. sp. strain 92 in MRS medium including 2 g/L laminaribiose or glucose in microaerobic conditions at 37 °C were set up in triplicate. Medium preparation and inoculation were performed as previously described. The cells were harvested after 4 and 12 hours of cultivation, respectively, as determined by the previously made growth curves to be in mid-log phase. RNA was immediately stabilised by adding RNAProtect Bacteria Reagent, cells were spun down and enzymatically lysed with lysozyme (Sigma, Merck, Kenilworth, New Jersey, United States), digested with Proteinase K (Qiagen, Valencia, CA, USA), and further disrupted mechanically and homogenised using a TissueLyser II (Qiagen) and 212–300 μm glass beads (Sigma, Merck). Total RNA was purified using an RNeasy mini kit including an on column DNase treatment according to the manufacturer’s protocol (Qiagen). RNA solutions were treated with TURBO-free DNase (Ambion, Life Technologies) to further remove gDNA contamination. RNA concentrations and purity were determined using a Nanodrop spectrophotometer (Nanodrop Technologies) and integrity was checked by gel electrophoresis. Quantitative PCR (qPCR) analysis was performed using the primers in Supplementary Table S3. 20 ng of complementary DNA (cDNA) template was used in each qPCR amplification, run in duplicate on the same plate. Detection of the PCR product was carried out by the CFX384 Real-Time PCR system (Biorad) using the DNA-binding dye SYBR Green I (SsoAdvanced, Biorad). To account for variation related to DNA input amounts or the presence of PCR inhibitors, two reference genes (*rpoB_730* and *ddl_1049*) were selected by GeNorm out of seven tested candidate reference genes (Supplementary Table S3).

## Supplementary Information

Supplementary Information.

## Data Availability

The data that support the findings of this study are available from the corresponding author upon reasonable request.
