# Supplementary material for: Taxogenomic assessment and genomic characterisation of Weissella cibaria strain 92 able to metabolise oligosaccharides derived from dietary fibres
Source: Sci Rep. 2020 Apr 3;10:5853. doi: 10.1038/s41598-020-62610-x (PMC7125115; doi:10.1038/s41598-020-62610-x)
Supplement: Supplementary file 1 — Supplementary Information. [file 41598_2020_62610_MOESM1_ESM.pdf]

Supplementary Information

Taxogenomic assessment and genomic characterisation of *Weissella cibaria* strain 92 able to metabolise oligosaccharides derived from dietary fibres

Anna Månberger[1,*], Phebe Verbrugghe[2], Elísabet Eik Guðmundsdóttir[3], Sara Santesson[1], Anne Nilsson[2], Guðmundur Óli Hreggviðsson[3,4], Javier A. Linares-Pastén[1,*] and Eva Nordberg Karlsson[1,*]

[1] Biotechnology, Department of Chemistry, Lund University, PO Box 124, SE-221 00 Lund, Sweden

[2] Food Technology, Engineering and Nutrition, Lund University, PO Box 124, SE-221 00, Lund, Sweden

[3] Matís, Vinlandsleid 12, IS-113, Reykjavík, Iceland

[4] Faculty of Life and Environmental Sciences, University of Iceland, Askja, IS-101, Reykjavík, Iceland

[*] Corresponding author, e-mail: anna.manberger@biotek.lu.se, javier.linares_pasten@biotek.lu.se, eva.nordberg_karlsson@biotek.lu.se, +46 222 36 73 (JALP), +46 222 46 26 (ENK)

Supplementary Table S1. Sequencing and trimming data of *Weissella* sp. strains. Coverage is calculated based on genome size determined after the assembly, see **Table 1**.

		**85**	**92**	**142**	**AV1**
Sequencing					
DNA Library	Reads passed filter	2 354 724	995 989	1 522 075	2 188 909
	Total Mbp	1 413	598	913	1 313
	Coverage	534	240	340	513
Mate pair library	Reads passed filter	-	1 226 160	1 429 182	1 612 465
	Total Mbp	-	613	715	806
	Coverage	-	232	287	300
Total coverage		534	472	627	813
Trimming and quality filtering					
DNA Library	Paired reads	1 531 549	846 854	932 023	1 433 933
	Unpaired reads	665 340	123 219	464 090	595 342
	Total Mbp	951	463	594	883
	Coverage	360	186	221	345
Mate pair library	Paired reads	-	637 683	765 720	793 512
	Unpaired reads	-	492 074	531 757	613 272
	Total Mbp	-	398	464	495
	Coverage	-	160	173	193
Total coverage		360	346	394	538

Supplementary Table S2. Screening of carbohydrate fermentation in *Weissella cibaria* strain 92. Carbohydrate fermentation was considered present (+) if the specific growth rate, μ, was at least 0.1 h^{-1} and the *p*H decreased at least 0.2 units over 48 h of cultivation. Otherwise, no or negligible carbohydrate fermentation (-) was assumed. P - production, U - uptake, n.d. - not detected. Production and uptake are relative to the amount of product that was within the medium at the start of cultivation.

**Carbohydrate**	**Carbohydrate fermentation**	**Fermentation products**				
		Lactate	Ethanol	Acetate	Propionate	Butyrate
1 mL cultivation – microaerophilic						
Glucose	+	P	P	U	n.d.	n.d.
Laminaribiose	+	P	P	P	n.d.	n.d.
Laminaritriose	-					
Laminaritetraose	-					
Arabinose	+	P	n.d.	P	n.d.	n.d.
Arabinobiose (A_2)	+	P	n.d.	P	n.d.	P
Arabinotriose (A_3)	-					
Arabinotetraose (A_4)	-					
Arabinopentose (A_5)	-					
N-Acetyl-glucosamine	+	P	P	P	n.d.	n.d.
Diacetyl-chitobiose	-					
75 mL cultivation – anaerobic						
Glucose	+	P	P	P	n.d.	n.d.
Arabinose	+	P	n.d.	P	n.d.	P
Xylose	+	P	n.d.	P	n.d.	P
Negative control	-					

Supplementary Table S3. Primer sequences used for real-time quantitative reverse transcription-PCR (RT-qPCR). peg refers to protein encoding gene from annotation.

Gene_peg	Forward primer	Reverse primer
Reference genes		
gyrA_402	TGACGACCAACAAGGGTGTA	TAAGCGAATAACCCGGACAC
gyrB_403	TTGATGTCACAAAGGCGAAG	ACAATGGACGCATGTAACGA
dnaG_1592	GCGTAATCTGTTTGGGCTGT	CCGCATGAGAAGCAGTGATA
rpoB_730	GTCCATCAATGGAGCAAGGT	TAAACATCATCGCGGATCAA
rpoC_731	TGGACACATCTTGGCACCTA	CCTTCACGACCAGCTTCTTC
rpoD_7593	GATGACACAGCCAAGCAAGA	ATTGCATACCACGACCAACA
ddl_1049	ATGCCATGGACAAGGAAAAG	CTTCCGCCACAATCGTATCT
Target genes		
bgl_197	GATACGGCGTTCAAGGATGT	AAATCGGCATCATAGCGTTC
bglB_302	TTGCGCATACCAAGTATCCA	TTACGCGCTGCATAGACATC
bglB_303+905	TCAATTGCGGATGTGATGTT	ATCCCACGGTGATTTGGATA
bglB_379	CCGTTATCAAAAGCCGTTGT	ATGTGCTCGCGGAAATAATC
bglB_461	CCGTTATGCGGAGACGTTAT	AATACCACCAGCGTTCCAAG
bglB_1258	TTTATGATTGGGGGCATGTT	TGCACGTCTGAGAACCAGTC
bglB_1261	TCCAATGCTGCAAAACTCAG	AATGCGGATGCCAATAACTC
bglB_1679	GCTGACGTTGAGGTTGTGAA	GTAACGCAAGCCTTGTGGAT
bglB_2328	TGTCGACCTGATGGGTTACA	CCCATAACGCTTCGACATTT
eng_661	GCAACGTTAAGCAAGCAACA	CGCAACTTCCTTGGCTTTAG
gh13_853	AATTGGCGCAGTATTTTTGG	CAGTGCTGGATTTTCCCAAT
bglB_303	CGCAAACACGGTTGATTATG	GTTGTAGGCGAGCCATTCAT

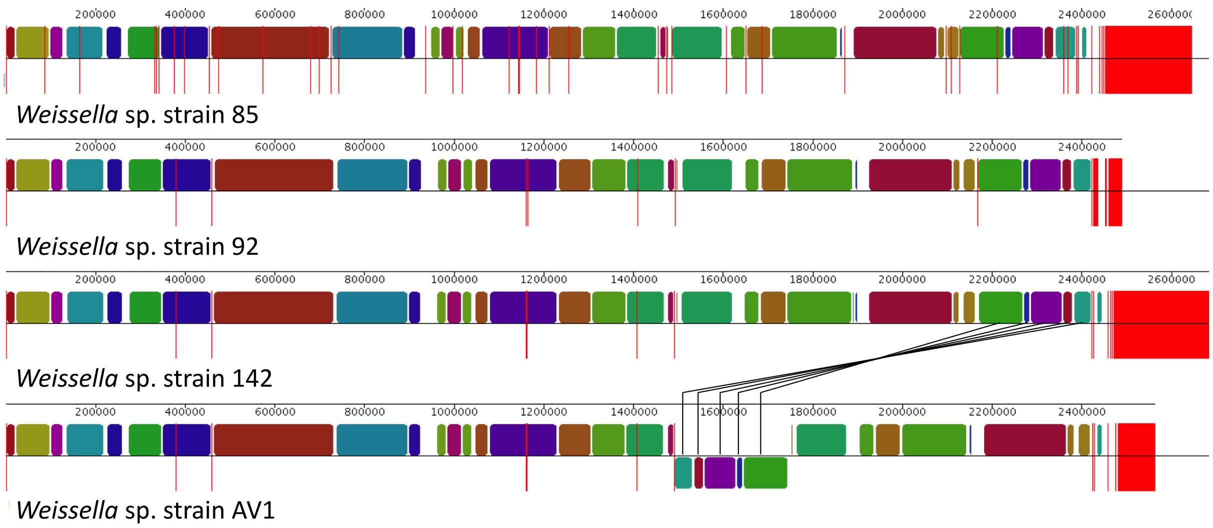

Supplementary Fig. S1. Alignments of the assembled genomes. The border of each contig is marked by a red line. Coloured blocks are regions aligned to regions in the other genomes, marked in the same colour. Blocks under the line indicate that the alignment is made in the reverse direction relative to the first genome (*Weissella* sp. strain 85). For a clearer picture, only major rearrangements of aligned regions (> 10.000 bp) are marked by connecting lines.

	**New *Weissella* sp. strains**			***Weissella cibaria***									***Weissella confusa***		
	85	AV1	142	FBL5	AB3b	CMU	KACC 11862	CMS3	CMS2	ff3PR	CH2	MG1	LBAE C39-2	DSM 20196	MBF8-1
W. sp. strain 92	100.0	100.0	100.0	99.9	99.9	99.8	99.8	99.8	99.8	99.7	99.7	99.6	88.0	87.4	86.6
W. sp. strain 85		100.0	100.0	99.9	99.9	99.8	99.8	99.8	99.8	99.7	99.8	99.6	88.0	87.3	86.6
W. sp. strain AV1			100.0	99.9	99.9	99.8	99.8	99.8	99.8	99.7	99.8	99.6	88.0	87.1	86.7
W. sp. strain 142				99.9	99.9	99.8	99.8	99.8	99.8	99.8	99.8	99.7	88.0	87.1	86.7
W. cibaria FBL5					99.9	99.9	99.9	99.9	99.9	99.7	99.7	99.4	88.7	87.9	88.5
W. cibaria AB3b						99.7	99.7	99.7	99.7	99.9	99.8	99.9	87.3	87.3	87.1
W. cibaria CMU							100.0	100.0	100.0	99.8	99.8	99.8	86.4	85.3	85.8
W. cibaria KACC 11862								100.0	100.0	99.9	99.9	99.9	86.3	85.3	86.0
W. cibaria CMS3									100.0	99.8	99.8	99.8	86.4	85.7	85.8
W. cibaria CMS2										99.8	99.8	99.8	86.4	85.3	86.0
W. cibaria ff3PR											99.9	100.0	87.4	87.9	86.1
W. cibaria CH2												99.9	88.6	87.5	87.1
W. cibaria MG1													87.1	87.5	86.8
W. confusa LBAE C39-2														99.9	99.9
W. confusa DSM 20196															99.4

- = 100
- ≥ 99.9, < 100
- ≥ 99.8, < 99.9
- ≥ 99.5, < 99.8
- ≥ 99.0, < 99.5
- ≥ 90, < 99
- ≥ 80, < 90
- ≥ 70, < 80
- ≥ 60, < 70
- ≥ 50, < 60

	Weissella species												
	sp. DD23	*confusa*	*parames-enteroides*	*hellenica*	*bombi*	*jogaejotgali*	*ceti*	*oryzae*	*kandleri*	*viridescens*	*minor*	*halotolerans*	*koreensis*
W. cibaria CH2	99.8	87.5	72.1	71.8	71.4	69.3	65.3	64.9	63.7	63.7	62.5	62.4	60.6
W. sp. DD23		88.3	74.2	74.1	71.9	68.1	70.5	68.1	58.8	67.1	59.4	65.7	63.8
W. confusa DSM 20196			72.9	69.2	76.6	71.4	66.3	68.8	65.1	66.8	61.3	66.6	56.7
W. paramesenteroides ATCC 33313				81.3	83.6	87.7	64.5	60.2	57.0	60.4	62.2	62.7	62.8
W. hellenica R-53116					92.5	84.7	62.6	62.4	68.8	62.6	60.3	63.1	62.0
W. bombi R-53094						82.0	65.8	67.5	61.4	67.2	67.6	65.2	64.3
W. jogaegotgali FOL01							54.4	59.3	56.5	56.6	65.2	57.7	59.8
W. ceti WS105								64.9	66.8	72.4	64.9	58.4	63.6
W. oryzae SG25T									64.6	59.3	61.7	59.3	66.1
W. kandleri DSM 20593										59.2	61.8	56.8	72.0
W. viridescens DSM 20410											78.6	65.1	56.0
W. minor DSM 20014												67.8	59.9
W. halotolerans DSM 20190													57.3

Supplementary Fig. S2. Sequence identity in percentage of bidirectional gene pairs from pairwise comparison of strains within *Weissella*.

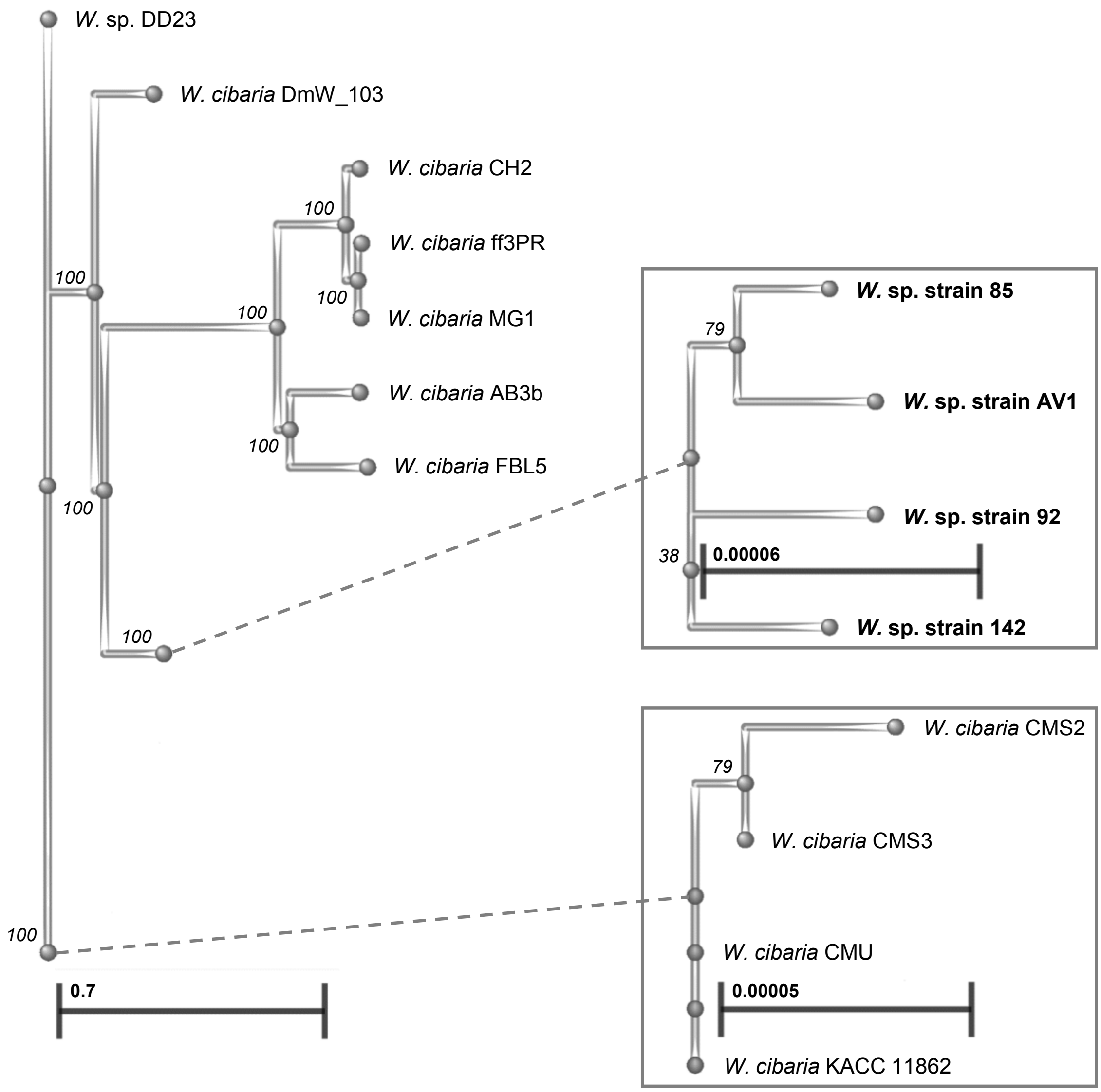

Supplementary Fig. S3. Core genome phylogeny of *Weissella cibaria* and *W.* sp. strains 85, 92, 142, AV1 and DD23 illustrated as an unrooted cladogram. The scales represent the genetic distance as number of substitutions divided by length of the sequences of the core genome of the group. The numbers adjacent to each branch node are the support values expressed as percentages.
